# “The ancient and the new”: is there an interaction between cytomegalovirus and SARS-CoV-2 infection?

**DOI:** 10.1186/s12979-020-00185-x

**Published:** 2020-05-27

**Authors:** Paul Moss

**Affiliations:** Institute of Immunology and Immunotherapy, University of Birmingham and Birmingham Health Partners, University Hospitals NHS Foundation Trust, Birmingham, UK

## Abstract

The SARS-CoV-2 pandemic represents one of the greatest infectious challenges to humanity in recent history. One of the striking features of infection is the heterogeneous clinical response with worse outcomes observed in older patients and those with underlying health conditions. To date the potential impact of previous infection history has been poorly investigated as a potential determinant of risk. Cytomegalovirus (CMV), a persistent herpesvirus infection whose prevalence increases with age, is a major modulator of immune function and several observations suggest that infection might act to influence clinical outcome following SARS-CoV-2 infection. In particular, CMV is associated with the acceleration of immune senescence and has been linked to a range of cardiovascular and metabolic disorders. This review addresses mechanisms by which cytomegalovirus infection may act to worsen the clinical outcome of SARS-CoV-2 infection, discusses how these potential links could be investigated, and assesses the potential significance of any findings that emerge.

## Introduction

The SARS-CoV-2 pandemic, which is believed to have originated in Wuhan in 2019, has already led to the deaths of over 340,000 people, a number that is rising steadily at this time [1]. Indeed, the virus represents one of the most important challenges to global health since Spanish Flu in 1918. At this stage, no effective treatment or vaccine is available and the mortality rate is estimated at around 2% [2]. One of the striking features of SARS-CoV-2 infection is that there is a very heterogeneous clinical outcome in different population groups. In particular, mortality risk is greatly increased in older people and also those with underlying health conditions such as cardiovascular disease, hypertension or diabetes. The explanation for these associations is unclear although a dysregulation in immune function with age (‘immune senescence’) is a well-established phenomenon. However, to date, the importance of previous infection history has received little interest as a potential determinant of clinical outcome. In particular, all adults harbour a range of persistent viral infections and this ‘virome’ plays an important role in promoting maturation of immune function and may also impact on the ability to generate immune responses to novel pathogens [3]. As such a primary infection with COVID-19 builds on an established platform of chronic infectious burden and this legacy may act as a determinant of outcome.

The herpesvirus family is one of the best characterized and largest group of persistent viral infections [4]. These eight viruses share a range of features including a relatively mild primary infection in most cases followed by lifelong persistence as a consequence of viral latency and sustained immunological control of viral replication. Cytomegalovirus (CMV) is the largest member of this family with a genome of 235 kb that encodes over 160 proteins. The clinical sequelae of CMV infection include a range of characteristic features and several of these would suggest that this virus, in particular, may act as a important influence on the clinical outcome of SARS infection. In this regard, any such association might be seen in either the extent of SARS-CoV-2 viral replication or the quality of the subsequent immune response. A secondary influence of the acute inflammation leading to enhanced CMV reactivation must also be considered.

### Cytomegalovirus

Cytomegalovirus is one of the most common persistent infections within the human population and it is likely that over 4 billion people are infected at the current time [5]. Infection is often encountered very early in life but may occur at any age and is usually asymptomatic. The virus then persists in a range of tissues including myeloid cells, vascular endothelium and renal tissue. Of note, the rates of CMV seropositivity (a marker of persistent infection) are very high in populations that have suffered high mortality rates from SARS-CoV-2 infection such as northern Italy, China and Spain [6]. In addition, infection rates are higher in people from lower socio-economic groups, a subset of the population that appears to have higher mortality rates from SARS-CoV-2 infection [7]. A striking feature of Covid-19 is the increased mortality rate in men compared to women and here it may be noteworthy that the influence of CMV on longer term health in women may be less significant than observed for men [8].

One of the characteristic and unique features of cytomegalovirus infection is its influence on the immune response. The virus acts as a hugely important influence on the maturation and long term composition of the immune repertoire [9, 10]. This is seen most clearly in the number and proportion of cytotoxic T and NK lymphocytes within the peripheral circulation which are increased by 30 to 40% in CMV-seropositive individuals [11–13]. Importantly this expansion in the number of virus-specific effector and memory cells is associated with a substantial decrease in the relative proportion of naive lymphocytes. Further associations include alterations in systemic inflammatory markers and infection of a proportion of myeloid cells. The significance of these findings in relation to the impact of SARS-CoV-2 infection on immune health are discussed below.

## “The smoking gun”: mechanisms by which CMV might be implicated in the clinical outcome of SARS-CoV-2 infection

### Immune senescence

Wikby and colleagues first identified CMV as component of the ‘immune risk phenotype’ associated with increased mortality in elderly individuals in 2002 [14]. This seminal finding triggered a number of additional epidemiological investigations, many of which confirmed this initial finding in different populations [15, 16]. However, it should also be noted that CMV infection alone has not been confirmed as an independent risk factor in all studies and there remains some debate about potential populations at risk [17]. The influence of CMV serostatus on vaccine responses in older people has also been investigated with conflicting findings in different studies [18, 19].

Somewhat surprisingly, CMV seropositive status has actually being shown to boost vaccine responses in younger people [20] and this is perhaps in keeping with its profound ability to drive maturation of Th1 immune responses in children following primary infection [21]. An emerging consensus is that CMV may act to enhance immune function in younger people but then becomes a negative impact on immune function in older people [22].

**Fig. 1:**
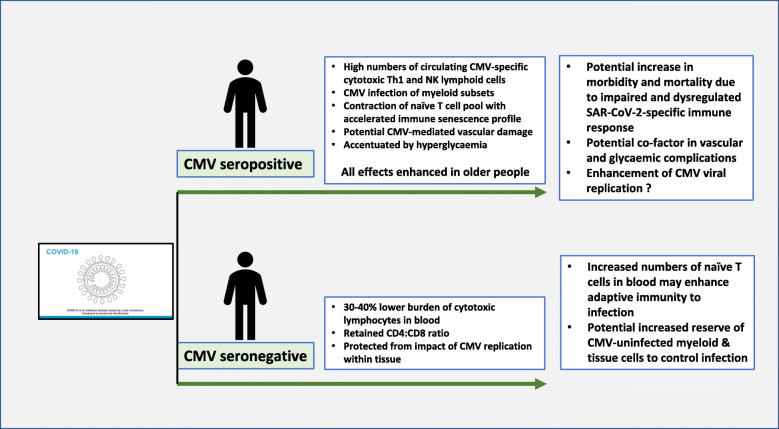
Schematic representation of the potential influence of CMV serostatus on the clinical outcome following SARS-CoV-2 infection. Drawing of coronavirus from WHO

As such, the profound correlation between risk of mortality from SARS-CoV-2 infection and increasing age might be explained partly by the negative impact of CMV on immune function in older people (Fig. 1). This effect may be driven by the phenomenon of ‘memory inflation’ which refers to the gradual accumulation of CMV-specific effector and memory cells during age [23]. Importantly, this is associated with a reduction in the naive T cell pool [11]. Indeed, it has been estimated that CMV infection accelerates attrition of the naive T cell pool by approximately 20 years and it is exactly this naive repertoire that will be required for generating adaptive immune responses against a novel virus such as SARS-CoV-2 [9]. Thus, the enormous host investment in CMV-specific immunity, at the expense of the naive repertoire, could act as a direct mechanism to limit protective immune responses against this novel infection. In addition to the magnitude of the adaptive immune response against SARS-CoV-2, CMV infection might also influence the quality of effector cells. This has already been shown in relation to CD4+ responses to heterologous viruses and immunogens in which the phenotypic profile of antigen-specific cells is influenced by CMV serostatus [24]. Other potential mechanisms may also be relevant. CMV infection of myeloid cells may modulate the profile of ‘macrophage activation’ that is seen in severe Covid-19 phenotypes. There has also been interest in the impact of CMV infection on the circulating serum proteome and this may also act to bias the nature of the immune response against infectious challenge.

### Cardiovascular disease

A characteristic feature of COVID-19 has been the significantly increased risk of mortality in patients with established cardiovascular disorders [25]. This includes hypertension, heart failure and ischemic heart disease. Again, these features could potentially be influenced by CMV serostatus at the time of infection. Cytomegalovirus can infect cells within the vascular endothelium and this appears to be an important site of intermittent viral replication [26]. It therefore makes perfect sense that the huge investment in CMV-specific T cells that circulate within the blood represents ‘immune surveillance’ of the vascular system [27]. CD4+ and CD8+ cytotoxic T cells are recruited rapidly to sites of viral activation where they suppress replication through cytokine release and cytotoxic activity [28]. It would therefore not be surprising if this process were to lead to vascular damage over many decades of infection.

These immunological findings underlie epidemiological studies which show a clear association between CMV serostatus and vascular disorders. This was noted in early studies within US populations [29, 30] and a cohort within England demonstrated a near two-fold increased risk of cardiovascular mortality in older patients who were CMV positive [31]. The mechanism underlying this correlation remains uncertain and there has been intense debate as to whether or not the virus can promote atheromatous vascular pathology. This remains uncertain, but it does appear that the virus can drive the development of arteriosclerosis, a process in which blood vessels become stiff and lose elasticity [32, 33]. This condition is associated with raised blood pressure and a number of reports have directly implicated CMV infection with increased blood pressure in both younger and older people, a finding that could easily explain the increased risk of cardiovascular death [34, 35].

These observations suggest that the intimate relationship between CMV infection and the vascular system may also act as a potential mechanism to enhance pathophysiological consequences of SARS infection.

### Diabetes

Finally, a somewhat surprising observation from the SARS-CoV-2 pandemic has been the significant increased clinical risk for patients with diabetes mellitus [36]. Dysregulation of glucose metabolism is frequently observed in patients with severe infection and as such the excess risk from diabetes may relate partly to this effect. Furthermore, diabetes is an established risk factor for vascular pathology and heart disease. However, it is also noteworthy that the magnitude of the CMV-specific T cell response is influenced by systemic glycemia. In a cohort study of 1103 individuals the number of effector T cells was strongly correlated with the concentration of HbA1c, a marker of glucose concentration over the preceding few weeks [37]. T cells with the typical phenotype of CMV-specific populations have also been correlated with vascular complications in diabetes. It is not clear if CMV infection predisposes directly to the development of diabetes or may act to accentuate tissue damage in the setting of established hyperglycaemia. The latter is more likely although CMV infection in mice does lead directly to increased systemic inflammation and hyperglycaemia [38]. Overall these observations again point to a potential interaction between CMV, hyperglycaemia and acute SARS-CoV-2 infection that may together promote tissue damage and increase clinical complications.

## How might the potential association between CMV and SARS-CoV-2 be investigated?

Not surprisingly, research into SARS-CoV-2 infection is currently the major priority for science communities worldwide. If persistent infection with viruses such as CMV do indeed impact on clinical outcome it will be important to consider what steps may be needed to investigate this.

-The first, and most definitive assessment, would be to determine the relative clinical outcome from SARS-CoV-2 infection in matched populations assessed by CMV serostatus. Such analysis would be straightforward and inexpensive and the only limitation is access to adequate numbers of serum samples from well-characterised clinical cohorts. These are now becoming available in many settings.

-Notwithstanding the outcome of this primary analysis, it would also be of interest to look at the titre of CMV-specific antibodies within CMV seropositive patients. It has been observed in many studies that patients with high titres of antibody display a range of impaired clinical outcomes although it is not clear if this is a ‘cause or effect’ in relation to secondary health conditions [39]. Indeed, CMV-specific antibody titres are increased following episodes of subclinical viral reactivation which are increased in episodes of stress, inflammation or intercurrent disease [40]. As such they are an indirect marker of poor health. However, cytomegalovirus-specific antibody titres are closely correlated with the cellular immune response against the virus [41]. As such it is also possible that the direct immunopathological consequences of elevated populations of CMV-specific cytotoxic cells can directly mediate health complications.

-If such serological studies do suggest an association between CMV infection and Covid-19 outcome then further analysis should be undertaken to assess how cytomegalovirus may act to modify SARS-CoV-2 viral load after infection or modulate the magnitude or functional profile of the SARS-CoV-2-specific immune response. In parallel, it will also be of interest to assess whether CMV reactivation is observed in patients with Covid-19. Indeed, other viruses may also be at play and some of the features of severe infection resemble hemophagocytic syndrome, itself commonly triggered by the herpesvirus Epstein-Barr Virus.

## Implications of a potential association between CMV and clinical outcome following Covid-19 outcome

If laboratory studies do confirm an interaction between CMV serostatus and the clinical outcome of Covid-19 infection it is important to reflect on how this might be utilised to modify the severity of the current pandemic. CMV-specific drugs could be used to suppress viral load if this were indeed elevated in severely ill patients. However, these would not reverse the immunomodulatory effects of CMV infection over the short term. CMV-specific memory cells have a half-life of many months [42] and antiviral therapy within mice is required for at least 6 or 12 months to improve functional immune responses [43]. A vaccine that protects against CMV infection is of high global importance, particularly for its ability to reduce congenital infection, but is unlikely to be available during the current pandemic. The most important insight may relate to defining individual risk from SARS-CoV-2 infection, something that could help to guide quarantine policy for ‘at risk’ groups.

It is now clearly established that the complexity of *Homo sapiens* is much greater than simply eukaryotic cells. The microbiome has taken centre stage in recent years and is certainly an important determinant of human health. The virome has received somewhat less attention although its impact may be no less considerable. At a time of global challenge from a new emerging virus it is timely to think how this may be modulated by carriage of an ancient and highly complex virus that has taken many millions of years to adapt to co-existence with primates. The control of SARS-CoV-2 infection cannot wait that long.

## Conclusions

- SARS-CoV-2 infection is associated with much higher rates of mortality in older people and those with coexisting health disorders. The underlying basis for this is not certain.
- Cytomegalovirus is a common and persistent herpesvirus infection and the proportion of people that are infected increases with age.
- Older people who are infected with cytomegalovirus have been shown to display accelerated immune senescence and increased death rates from vascular disease.
- Cytomegalovirus may act as a negative risk factor for clinical outcome following SARS-CoV-2 infection and this could be of importance in relation to clinical management and optimisation of epidemiological control strategy.

## Data Availability

Not applicable.

## Abbreviations

SARS-CoV-2: Severe acute respiratory syndrome coronavirus 2
CMV: Cytomegalovirus
